# Items of Interest

**Published:** 1895-01

**Authors:** 


### ITEMS OF INTEREST.

CANADA seems peculiarly unfortunate of late. A cablegram from Antwerp, Belgium, announces that a cargo of sheep suffering from foot and mouth disease, has just been detained at that port of entry, and are likely to be returned to place of shipment in Canada.

There is a disposition to discard gray and white horses in the United States Army. A similar action, we believe, has also been adopted by the German Army.

The first biennial report of the State Veterinary Medical Board of California, covering the period from March, 1893, to November, 1894, has just been received. Some nine meetings of the board were held alternately at San Francisco and Los Angeles, examining in all fifty-one diplomas of graduates, all of which were found genuine, and licenses granted to register in their respective counties. Some fifty-one non-graduates presented themselves for examination, twenty-four of whom passed satisfactory examinations and twenty-seven were rejected. It will be remembered that this act only applies to cities and towns of two thousand or more inhabitants.

Harvard Veterinary Department has just done away with the subscription plan of treatment in its veterinary hospital, which will be grateful news to the entire profession. These means of obtaining hospital practice have always been distasteful to the local profession wherever they existed, and have caused many serious splits in the ranks. It is to be hoped that other schools may soon see their way to do likewise, and thus heal other breaches and regain the undivided support and assistance of the profession in the cities where schools exist.

Texas has just quarantined against the shipment of cattle from Mexico, owing to a widespread and serious outbreak of splenic fever now prevailing in that country.

No less than eight horses, two mules, and two hogs were Philadelphia's record in one day of electrocutions from broken telephone wires coming in contact with trolley and electric light wires.

Dr. Alex. Glass, lecturer on canine pathology at the Veterinary Department of the University of Pennsylvania, is busily engaged in translating the work of Prof. Mueller, of Germany, on canine pathology. This will be a very grateful addition to English-speaking and reading veterinarians.

The Philadelphia Board of Health, which governs the milk supply of Philadelphia, has just given its unqualified support and indorsement to the proposed act for the establishment of a Live Stock Sanitary Commission in Pennsylvania. Many letters from prominent farmers, dairymen, and stock raisers approving the act have already been received.

Newspaper warfare against sanitary measures in the shape of unsigned letters of attack, or by some sign covering up the writer's name, should have little weight in influencing legislation.

Mr. J. E. Gillingham, one of the veterinary school's benefactors in Pennsylvania, and one of the first stock raisers to rid his herd of tuberculosis, has written a strong letter urging the passage of the proposed live stock sanitary commission in this State.

In various centres of our country there is now being agitated the wisdom and necessity of making horse meat an article of commerce on our public stalls, and why not? He is in every sense cleaner than the hog or duck.

The North Dakota Veterinary Medical Association has strengthened its membership by the addition of seven new members at its recent meeting held in November.

The Philadelphia *Ledger* of January 1st contained a two-column report on the merits of horse flesh as a food apropos of the recent horse-flesh dinner given at Newark, N. J., by Dr. J. D. Hopkins. Many and varied were the opinions given by those interviewed, among whom might be mentioned Drs. Salmon, Harger, Gadsden, Weber, Glass, Zuill, Bridge, and E. Mayhew Michener. Mrs. S. T. Rorer, the famous cooking expert, refers to its lack of quality and toothsome character compared with beef. It looks now as if it may soon be a recognized and admitted article of merchandise on our stalls.

Very earnest work is now being done by the Army Legislative Committee of the U. S. V. M. A. toward that long-wished-for goal—official recognition of veterinarians in army service.

